# Melatonin Inhibits Lipopolysaccharide-Induced Inflammation and Oxidative Stress in Cultured Mouse Mammary Tissue

**DOI:** 10.1155/2019/8597159

**Published:** 2019-02-12

**Authors:** Guang-Min Yu, Wen Tan

**Affiliations:** Institute of Biomedical and Pharmaceutical Sciences, Guangdong University of Technology, Guangzhou 510006, China

## Abstract

To determine whether melatonin can protect cultured mouse mammary tissue from lipopolysaccharide- (LPS-) induced damage, we investigated the effects of melatonin on the mRNA and protein levels of proinflammatory cytokines and chemokines in LPS-stimulated mammary tissue *in vitro*. This study also examined the IgG level in both cultured mammary tissue and the culture medium. In addition, we investigated the potential benefits of melatonin on the expression of antioxidant relative genes following LPS treatment in cultured mammary tissue and evaluated ROS level in the culture medium. The results demonstrate that melatonin inhibited the mRNA expression of *TNF-α*, *IL-1β*, *IL-6*, *CXCL1*, *MCP-1*, and *RANTES* and the production of these cytokines and chemokines and IgG in LPS-stimulated mouse mammary tissue *in vitro*. In addition, melatonin increased *Nrf2* but decreased *iNOS* and *COX-2* mRNA expression after LPS stimulation. Similarly, the decreased level of dityrosine in the culture medium was increased by treatment with melatonin, while increased nitrite level was suppressed. This study confirms that melatonin inhibited LPS-induced inflammation and oxidative stress in cultured mouse mammary tissue. It might contribute to mastitis therapy while treating antibiotic resistance.

## 1. Introduction

Mastitis develops in 5–33% of women at some point during lactation [1]. It is important for two main reasons [2]. Firstly, mastitis reduces milk production and alters the cellular composition of milk. Secondly, it destroys local defenses within the breast itself. Mastitis is also an enormous risk factor causing vertical transmission of infections [3]. The condition may even develop to give rise to a local abscess [4].

Gram-negative bacteria are the most frequent causative agents of mastitis [5]. Lipopolysaccharide (LPS) is considered to be an important risk factor for the mammary cell inflammation [6]. It has been reported that inflammatory cytokines and chemokines play an important role in mastitis pathogenesis [7, 8]. The levels of these cytokines and chemokines increased in Gram-negative bacteria infection and LPS-infused mammary glands [9], which induces the migration of leukocytes [10] and the production of acute-phase proteins [11]. Following an inflammatory stimulus, reactive oxygen species (ROS) were produced and cause the cells damage [12].

Melatonin was first isolated from bovine pineal gland more than half a century ago [13]. It is the main chronobiotic hormone that regulates the circadian rhythms and seasonal changes in vertebrate physiology via its daily nocturnal increase in the blood [14]. The remarkable functional versatility of melatonin is reflected in its wide distribution within phylogenetically distant organisms including bacteria, protista, invertebrates and vertebrates, algae, plants, and fungi and is also found in various edibles, such as vegetables, fruits, seeds, and seafood [15, 16]. Melatonin shows multiple biological functions, such as antioxidant [17, 18], oncostatic [19], antiaging [20], chronobiotic actions [21], female reproduction [22, 23], innate immunity [24, 25], abiotic stress resistance [26], anticancer [27, 28], and antiradiative effects [29].

Previous studies have shown the ability of melatonin to repress proinflammatory cytokine and chemokine level and reduce oxidative stress in several experimental inflammations, including mastitis models [30–32]. Difference from the *in vivo* experiments and purified cells, the novelty of the present study is that we investigated LPS-stimulated mouse mammary tissue *in vitro*, which we try to mimic the local immunity. A series of experiments were then conducted to investigate the effect of melatonin under this situation. We evaluated the effects of melatonin on the mRNA and protein levels of proinflammatory cytokines and chemokines after LPS treatment. This study also examined the IgG level in both cultured mammary tissue and the culture medium. In addition, we investigated the potential benefits of melatonin on the expression of antioxidant relative genes following LPS treatment in cultured mammary tissue and evaluated ROS level in the culture medium.

## 2. Materials and Methods

### 2.1. Animals

Sea:ICR mice (8 wk old) were fed with standard laboratory chow and water *ad libitum* and kept under controlled conditions of temperature (24 ± 1°C), relative humidity (40–80%), and light (16 h light : 8 h dark cycle). Mice were treated in accordance with the regulations of SAC/TC 281 for animal experiments. Mice were placed into groups, with one male and one female as a couple. Eight days after the birth of the litter (a litter of at least 6 pups is required for adequate lactation) [33], the female mice were euthanatized by decapitation under anesthesia with intraperitoneal administration of pentobarbital sodium (Somnopentyl; Kyoritsu Seiyaku Co., Tokyo, Japan).

### 2.2. Tissue Culture

Mouse mammary tissue was prepared as previously described [34]. Briefly, about 2 g of mammary tissue was minced into small pastes (approximately 1 mm cubes). It was centrifuged three times at 250 *g* for 5 min each time. The small pieces of mammary gland were suspended in four 36 mm culture plates, each containing about 125 mg tissue and 2.5 mL DMEM/F12 containing 15% KnockOut™ serum replacement (Gibco BRL), 1% nucleosides (Millipore Co., Billerica, MA, USA), 1% nonessential amino acids (Gibco BRL), 1 mM sodium pyruvate (Gibco BRL), and 1% antibiotic-antimycotic mixed stock solution (Nacalai Tesque Inc., Kyoto, Japan). They were cultured at 37°C with 5% CO_2_. Melatonin (5 mg) was dissolved in 100 *μ*L of ethanol as the stock solution. In this study, melatonin and LPS (ALX-581-014-L001; Enzo Life Sciences, Farmingdale, NY, USA) were added into DMEM/F12 culture medium simultaneously to give final concentrations of 10 *μ*g/mL [31, 32] and 100 ng/mL [30], respectively. Mammary tissue was cultured for 6 h. Melatonin-untreated tissue containing ethanol as the solvent at the same concentration (0.08%, *w*/*w*) used in the tests were used as a negative control.

### 2.3. qRT-PCR

Total RNA was extracted using NucleoSpin RNA (Macherey-Nagel Inc., Düren, Germany) according to the manufacturer's protocol. It was then quantified by measuring absorbance at 260 nm and reverse transcribed by ReverTra Ace (Toyobo Co. Ltd., Osaka, Japan). Quantitative RT-PCR (qRT-PCR) was performed by KOD SYBR qPCR Mix (Toyobo Co. Ltd.) on Applied Biosystems StepOne Real-Time PCR System (Life Technologies Co., Darmstadt, Germany). The qPCR mixture consisted of 10 *μ*L KOD SYBR qPCR Mix, 0.5 *μ*M forward and reverse primers, 0.4 *μ*L ROX Reference Dye, 2 *μ*L template, and ddH_2_O was added to a total volume of 20 *μ*L. The parameters were as follows: initial denaturation at 98°C for 2 min; followed by 40 cycles of denaturation at 98°C for 10 s, annealing at 55–64°C for 10 s, and extension at 68°C for 1 min; melting curve from 60 to 99°C, increasing in increments of 0.5°C every 5 s. Normalization was performed using the housekeeping gene (*L19*) as a control. Primer sequences are listed in Table 1. Relative mRNA expression was calculated by the 2^–ΔΔct^ method. In addition, qPCR products were resolved on 2% (*w*/*v*) agarose gels containing 0.025% (*w*/*v*) ethidium bromide and sequenced for verification.

### 2.4. Western Blot Analysis

Protein sample from cultured mouse mammary tissue was prepared by homogenization in whole tissue extract buffer and then diluted by the same volume of 2×SDS sample buffer [37]. Equal amounts of protein (20 *μ*g/lane) were resolved by SDS polyacrylamide gel (10%) electrophoresis and transferred to Immobilon-P nylon membrane (Millipore Co.). The membrane was blocked in Tris-buffered saline and Tween 20 (TBST: 10 mM Tris, pH 7.5; 150 mM NaCl; and 0.05% Tween 20), containing 5% (*w*/*v*) nonfat Carnation instant milk (Nestle, Solon, OH). Blot was incubated with primary antibody (1 : 10,000 of anti-*β*-actin antibody, AC74, Sigma-Aldrich Chemical Co., St. Louis, MO, USA) overnight at 4°C. After washing in TBST, it was incubated with horseradish peroxidase- (HRP-) conjugated secondary antibody (1 : 5000 of donkey anti-mouse IgG, Sigma-Aldrich Chemical Co.) for 1 hour at room temperature. Then, it was visualized by using enhanced chemiluminescence Western blotting detection reagents (Amersham Pharmacia Biotech, Newark, NJ, USA) and appropriate exposure to X-ray film (Kodak, Rochester, NY, USA). Specific bands were quantified by densitometric analyses using a GelPro analyzer (Media Cybernetics, Bethesda, MD, USA).

### 2.5. ELISA

Culture medium was precleared by centrifugation at 3000 *g* for 20 min and stored at −20°C. The level of inflammatory cytokines (TNF-*α*, IL-1*β*, and IL-6) and chemokines (CXCL1, MCP-1, and RANTES) in duplicate samples of culture medium was measured with competitive enzyme-linked immunosorbent assay (ELISA) kits, according to the manufacturer's instructions (R&D Systems Inc., Minneapolis, MN, USA). The details of sensitivity and coefficient variation are listed in Table 2. The attenuance of the microplate was read at 450 nm.

The level of dityrosine in duplicate samples of tissue culture medium was determined by a competitive ELISA kit (JaICA, Shizuoka, Japan), according to the manufacturer's instructions. It was calculated with reference to a standard curve that typically ranged from 0.05 to 12 *μ*M.

### 2.6. The Griess Reaction

Nitrite concentration in the culture medium was measured using a nitrite colorimetric assay kit (Dojindo, Tokyo, Japan) based on the Griess reaction. It was determined by spectrophotometric analysis at 570 nm (Model 680 microplate 176 reader S/N 22002; Bio-Rad Laboratories, Hercules, CA, USA) with reference to a standard curve.

### 2.7. Statistics Analysis

Statistical analyses were carried out by one-way ANOVA followed by Duncan's multiple-range test (StatView; Abacus Concepts Inc., Berkeley, CA, USA). Data are expressed as mean ± S.D. of 6 independent experiments. *P* < 0.05 was considered statistically significant.

## 3. Results

**Figure 1:**
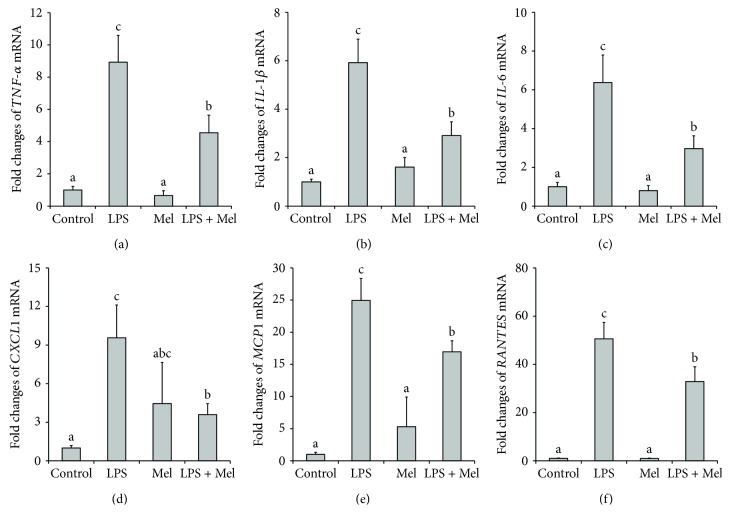
Effect of melatonin on relative mRNA level of inflammatory cytokines and chemokines in LPS-stimulated mouse mammary tissue. (a) Effect of melatonin on relative mRNA level of *TNF-α*. (b) Effect of melatonin on relative mRNA level of *IL-1β*. (c) Effect of melatonin on relative mRNA level of *IL-6*. (d) Effect of melatonin on relative mRNA level of *CXCL1*. (e) Effect of melatonin on relative mRNA level of *MCP-1*. (f) Effect of melatonin on relative mRNA level of *RANTES*. Control: containing ethanol as the solvent at the same concentration used in the tests. LPS: a working concentration 100 ng/mL LPS. Mel: a working concentration 10 *μ*g/mL melatonin. LPS+Mel: final concentrations of 100 ng/mL LPS and 10 *μ*g/mL melatonin. Values with different letters differ significantly (*P* < 0.05).

LPS caused substantial increase in the mRNA level of the inflammatory cytokines *TNF-α*, *IL-1β*, and *IL-6* in cultured mouse mammary tissue (*P* < 0.05, Figures 1(a)–1(c)). Addition of melatonin significantly decreased *TNF-α*, *IL-1β*, and *IL-6* mRNA level after LPS stimulation (*P* < 0.05). With regard to the inflammatory chemokines, LPS treatment markedly increased *CXCL1*, *MCP-1*, and *RANTES* mRNA level (*P* < 0.05, Figures 1(d)–1(f)); melatonin clearly suppressed the increase in *CXCL1*, *MCP-1*, and *RANTES* mRNA expression after LPS treatment (*P* < 0.05).

**Figure 2:**
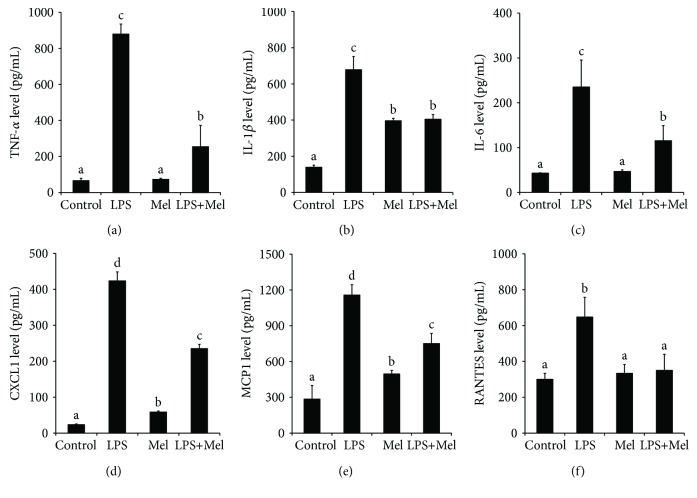
Effect of melatonin on inflammatory cytokine and chemokine expression in LPS-stimulated mouse mammary tissue. (a) Effect of melatonin on TNF-*α* expression. (b) Effect of melatonin on IL-1*β* expression. (c) Effect of melatonin on IL-6 expression. (d) Effect of melatonin on CXCL1 expression. (e) Effect of melatonin on MCP-1 expression. (f) Effect of melatonin on RANTES expression. Control: containing ethanol as the solvent at the same concentration used in the tests. LPS: a working concentration 100 ng/mL LPS. Mel: a working concentration 10 *μ*g/mL melatonin. LPS+Mel: final concentrations of 100 ng/mL LPS and 10 *μ*g/mL melatonin. Values with different letters differ significantly (*P* < 0.05).

LPS stimulation significantly increased the production of the inflammatory cytokines TNF-*α*, IL-1*β*, and IL-6 in the culture medium (*P* < 0.05, Figures 2(a)–2(c)). When present at melatonin, LPS-stimulated TNF-*α*, IL-1*β*, and IL-6 level was markedly suppressed (*P* < 0.05). In addition, the LPS-induced increase in chemokines (CXCL1, MCP-1, and RANTES) was significantly inhibited by melatonin (*P* < 0.05, Figures 2(d)–2(f)). Surprisingly, the administration of melatonin also significantly increased the basic level of IL-1*β*, CXCL1, and MCP-1 (*P* < 0.05, Figures 2(b)–2(e)).

**Figure 3:**
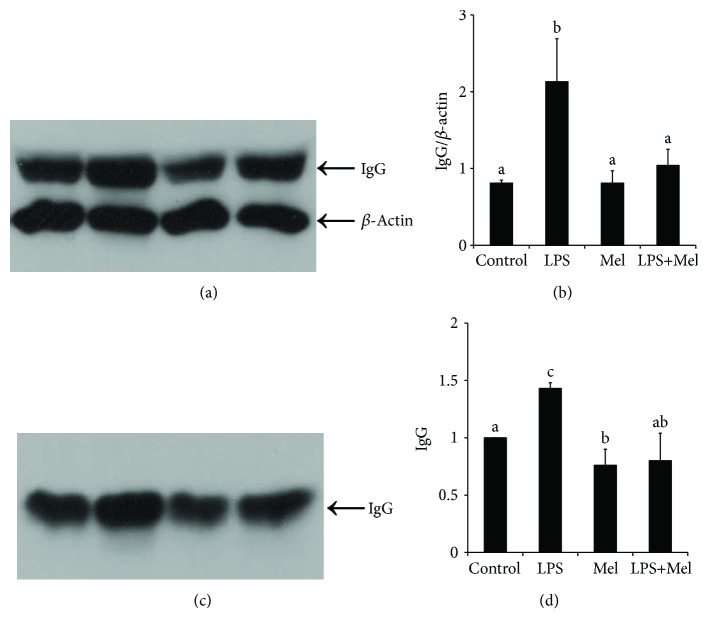
Effect of melatonin on IgG level. (a) Effect of melatonin on the expression of IgG in LPS-stimulated mouse mammary tissue. IgG protein was analyzed by Western blotting. (b) Relative abundance of IgG protein in LPS-stimulated mouse mammary tissue. Proteins were normalized with *β*-actin. (c) Effect of melatonin on the production of IgG in tissue culture medium. IgG protein was analyzed by Western blotting. (d) Relative abundance of IgG protein in tissue culture medium. Control: containing ethanol as the solvent at the same concentration used in the tests. LPS: a working concentration 100 ng/mL LPS. Mel: a working concentration 10 *μ*g/mL melatonin. LPS+Mel: final concentrations of 100 ng/mL LPS and 10 *μ*g/mL melatonin. Values with different letters differ significantly (*P* < 0.05).

Results of Western blot show that LPS significantly increased the protein level of IgG in cultured mammary tissue and the culture medium (*P* < 0.05, Figure 3). Both of them were markedly inhibited by melatonin administration (*P* < 0.05).

**Figure 4:**
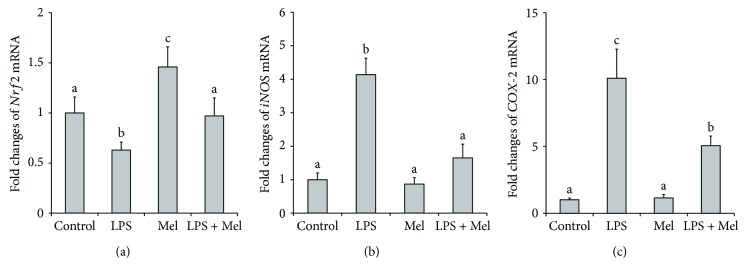
Effect of melatonin on relative mRNA level of antioxidant relative genes in LPS-stimulated mouse mammary tissue. (a) Effect of melatonin on relative mRNA level of *Nrf2*. (b) Effect of melatonin on relative mRNA level of *iNOS*. (c) Effect of melatonin on relative mRNA level of *COX-2*. Control: containing ethanol as the solvent at the same concentration used in the tests. LPS: a working concentration 100 ng/mL LPS. Mel: a working concentration 10 *μ*g/mL melatonin. LPS+Mel: final concentrations of 100 ng/mL LPS and 10 *μ*g/mL melatonin. Values with different letters differ significantly (*P* < 0.05).

As shown in Figure 4(a), LPS significantly inhibited the level of *Nrf2* mRNA in cultured mammary tissue (*P* < 0.05). Addition of melatonin eliminated this inhibiting effect. While the level of *iNOS* and *COX-2* mRNA was substantially increased by LPS stimulation (*P* < 0.05), melatonin significantly suppressed these effects (*P* < 0.05, Figures 4(b) and 4(c)).

**Figure 5:**
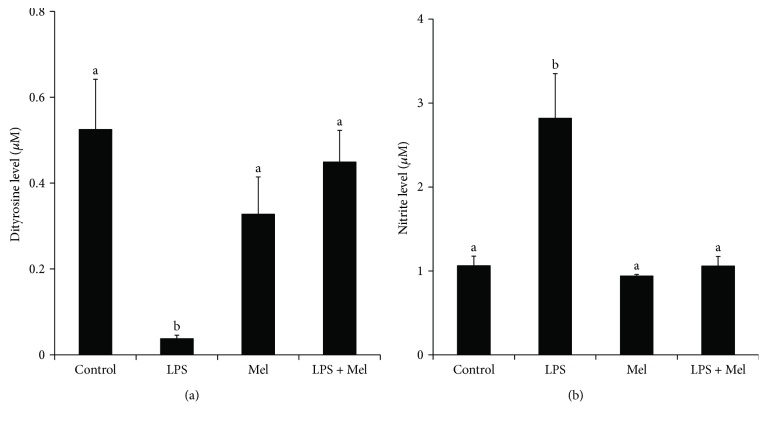
Effect of melatonin on the level of oxidative stress in tissue culture medium. (a) Effect of melatonin on the production of dityrosine. (b) Effect of melatonin on the production of nitrite. Control: containing ethanol as the solvent at the same concentration used in the tests. LPS: a working concentration 100 ng/mL LPS. Mel: a working concentration 10 *μ*g/mL melatonin. LPS+Mel: final concentrations of 100 ng/mL LPS and 10 *μ*g/mL melatonin. Values with different letters differ significantly (*P* < 0.05).

To evaluate oxidative stress, the level of dityrosine in the culture medium was determined by ELISA; it was substantially decreased by LPS stimulation (*P* < 0.05), but this effect was inhibited by treatment with melatonin (Figure 5(a)). The nitrite level in the supernatants of treated cultured mammary tissue was measured by the Griess reaction. Result showed that LPS markedly increased the production of nitrite (*P* < 0.05, Figure 5(b)). As expected, melatonin significantly suppressed the increase in nitrite level (*P* < 0.05).

## 4. Discussion

Since antibiotics have been used so widely and for so long, antibiotic resistance has become a major public health threat and it is growing [38]. Some beneficial effects of the currently used alternatives to antibiotics, i.e., probiotics [39], prebiotics [40, 41], organic acids [42], and phytogenics [43] on health have been well studied. In recent years, the anti-inflammatory effect of melatonin has been highly focused on [30–32]. The present study confirms that melatonin inhibited production of proinflammatory cytokines, chemokines, and IgG in LPS-stimulated mouse mammary tissue *in vitro*. In addition, results show that LPS-induced oxidative stress was also diminished by melatonin.

Previous studies attest to the anti-inflammatory activity of melatonin both *in vivo* and *in vitro*. Melatonin inhibited *IL-6* mRNA expression and diminished oxidative damage after venous infusion of LPS and peptidoglycan in rats [44] and also reduced the levels of TNF-*α*, IL-1*β*, and oxidative stress mediators in different regions of rat brains after intracerebroventricular administration of LPS [45]. In pregnant mice, melatonin suppressed LPS-induced increases in TNF-*α* level in the maternal serum and fetal brain [46]. In addition, melatonin reduced TNF-*α*, IL-1*β*, IL-6 levels and decreased the number of apoptotic neurons after intraventricular *Klebsiella pneumoniae* injection in rats [47]. Further, it has been observed that the expression of *TNF-α*, *IL-1β*, *IL-6*, and *IL-8* mRNA in LPS-stimulated RAW264.7 cells was inhibited by melatonin treatment [48]. Beneficial effects were also observed when melatonin was administered to LPS-stimulated granulosa cells of the quail *in vitro*. In that case, melatonin decreased LPS-induced mRNA expression of *IL-1β*, *IL-6*, *IL-8*, and suppressed oxidative stress level [32].

With regard to the mechanism of anti-inflammation, melatonin modulated TLR4-mediated inflammation through MyD88- and TRIF-dependent signaling pathways in LPS-stimulated RAW264.7 cells [48]. Melatonin also inhibited matrix metalloproteinase-9 activation in the LPS-stimulated RAW264.7 and BV2 cells by decreasing NF-*κ*B binding activity and translocation [49]. Focusing on mastitis model, a pretty important work has been done by Boulanger et al. [50]. They used a mammary epithelial cell line to evaluate melatonin on bovine neutrophil-induced oxidative stress following LPS stimulation. It is reported that melatonin could also inhibit LPS-induced inflammatory responses by activating peroxisome proliferator-activated receptor-*γ* in mouse mammary epithelial cells [30]. A recent study conducted by the authors indicates that melatonin suppressed the LBP–CD14–TLR4 signaling pathway in bovine mammary epithelial cells, which attenuated the LPS-stimulated increase in proinflammatory cytokines, chemokines, and positive acute-phase proteins at mRNA level [31]. The results of the present study proved that melatonin decreased the level of oxidative stress and the production of proinflammatory cytokines, chemokines, and IgG in LPS-stimulated mouse mammary tissue *in vitro*, which mimicked the local immunity.

It is interesting to notice that besides the effect that melatonin inhibiting the LPS-induced increases in proinflammatory cytokines and chemokines, it also significantly increased the basal level of some of them, such as IL-1*β*, CXCL1, and MCP-1. Results in this study are consistent with some previous studies that reported effects of melatonin under basal and immunosuppressed conditions *in vivo* [51, 52]. In fact, as an immune modulator, melatonin can exert both pro- and anti-inflammatory effects, which seems to largely depend on the cells and systems studied, and especially to the grade of inflammation. The proinflammatory effect seems to be observed under basal condition, whereas the anti-inflammatory effect is observed in the presence of high-grade inflammation [53]. Thus, melatonin appears to act as a buffer, allowing the immune system to respond to infections while attenuating serious damage in high-grade inflammation [16]. It is also thought that melatonin may promote the early stages of inflammation but suppress the sustained response to prevent chronic inflammatory disease [54].

## 5. Conclusions

The present study confirms that melatonin inhibited production of TNF-*α*, IL-1*β*, IL-6, CXCL1, MCP-1, RANTES, and IgG in LPS-stimulated mouse mammary tissue *in vitro*. In addition, melatonin increased *Nrf2* but decreased *iNOS* and *COX-2* mRNA expression after LPS stimulation. Similarly, the decreased level of dityrosine in the culture medium was increased by treatment with melatonin, while increased nitrite level was suppressed. Generally, melatonin inhibited LPS-induced inflammation and oxidative stress in cultured mouse mammary tissue, which mimicked the local immunity.

## Figures and Tables

**Table 1:** Primers used for qPCR.

Genes	Primer sequence (5′–3′)	Product size (bp)^1^	Tm (°C)
*L19*	Forward: CTGAAGGTCAAAGGGAATGTG	196	60
	Reverse: GGACACAGTCTTGATGATCTC		
*TNF-α*	Forward: AGTCCGGGCAGGTCTACTTT	422	60
	Reverse: GCACCTCAGGGAAGAATCTG		
*IL-1β*	Forward: GGAATGACCTGTTCTTTGAAGTT	345	60
	Reverse: GGCTCCGAGATGAACAACAAAA		
*IL-6*	Forward: CCGGAGAGGAGACTTCACAG	421	60
	Reverse: GGAAATTGGGGTAGGAAGGA		
*CXCL1*	Forward: AAGATGCTAAAAGGTGTCCCCA	389	55
	Reverse: CTCCCACACATGTCCTCACC		
*MCP-1*	Forward: GGTCCCTGTCATGCTTCTGG	236	64
	Reverse: CCTTCTTGGGGTCAGCACAG		
*RANTES*	Forward: ATATGGCTCGGACACCACTC	242	62
	Reverse: GGGAAGCGTATACAGGGTCA		
*Nrf2*	Forward: CTTTAGTCAGCGACAGAAGGAC	227	62
	Reverse: AGGCATCTTGTTTGGGAATGTG		
*iNOS*	Forward: GCATGGACCAGTATAAGGCAAGAC	222	64
	Reverse: GCATGGACCAGTATAAGGCAAGAC		
*COX-2*	Forward: AGACATCCTGATCCTGGTTT	197	60
	Reverse: GTTCAATGGGCTGGAAGACA		

^1^SYBR qPCR mix allows amplicon size of products in the present study [35, 36]. Abbreviation: *L19*: ribosomal protein L19 (housekeeping gene); *TNF-α*: tumor necrosis factor-*α*; *IL*: interleukin; *CXCL*: chemokine CXC motif ligand; *MCP-1*: monocyte chemotactic protein 1; *RANTES*: regulated upon activation, normal T-cell expressed and secreted; *Nrf2*: nuclear factor E2-related factor; *iNOS*: inducible nitric oxide synthase; *COX-2*: cyclooxygenase-2.

**Table 2:** The sensitivity and coefficient variation of ELISA kits.

Names	Sensitivity (pg/mL)	Assay range (pg/mL)	Intra-assay CV (%)	Interassay CV (%)
TNF-*α*	7.21	10.9–700	2.7–3.1	6.2–8.8
IL-1*β*	4.8	12.5–800	3–7.5	5.7-8.4
IL-6	1.8	7.8–500	3.5-6.7	6.1–8.9
CXCL1	2	15.6–1000	3.1–5.4	3–9.8
MCP-1	2	15.6–1000	5.1–8.3	4.6-7.3
RANTES	2	7.8–500	1.8–3.7	5.1–8

## Data Availability

The data used to support the findings of this study are available from the corresponding author upon request.
